# Association between the quality of care and continuous maternal and child health service utilisation in Angola: Longitudinal data analysis

**DOI:** 10.7189/jogh.13.04073

**Published:** 2023-08-11

**Authors:** Ai Aoki, Keiji Mochida, Michiru Kuramata, Toru Sadamori, Pedro Sapalalo, Lino Tchicondingosse, Olukunmi Omobolanle Balogun, Hirotsugu Aiga, Ketha Rubuz Francisco, Kenji Takehara

**Affiliations:** 1 Department of Health Policy, National Center for Child Health and Development, Tokyo, Japan; 2 TA Networking Corp., Tokyo, Japan; 3 Department of Global Health, Graduate School of Health Sciences, University of the Ryukyus, Okinawa, Japan; 4 Samauma Consulting LLC, Chiba, Japan; 5 Domus Custodius (SU) Lda. Tchikos Agency, Luanda, Angola; 6 School of Tropical Medicine and Global Health, Nagasaki University, Nagasaki, Japan; 7 Human Development Department, Japan International Cooperation Agency, Tokyo, Japan; 8 National Directorate of Public Health, Ministry of Health, Luanda, Angola

## Abstract

**Background:**

Many low- and middle-income countries (LMICs) prioritise minimising maternal, neonatal, and infant mortality. To improve maternal and child health, various evidence-based interventions have been introduced. Quality of care is pertinent while strengthening service utilisations. Achieving optimal-quality care is often marred with difficulties, such as inadequate skills and knowledge of health workers, poor fidelity to protocols, and poor user acceptance. Angola is a LMIC facing these problems. This study aimed to demonstrate the influence of health facilities’ quality of care at antenatal care (ANC) on subsequent maternal, newborn and child health (MNCH) service utilisation in Angolan pregnant women.

**Methods:**

Population-based cohort data from the Maternal and Child Health Handbook (MCH-HB) effectiveness study were analysed. The original study was conducted among women who became pregnant between March and April 2019 in Benguela Province, Angola. Socioeconomic and MNCH service utilisation indicators were collected through interviewer-administered structured questionnaires. The indicator of quality of care was a composite measure that assessed the implementation of the MCH-HB based on the RE-AIM framework, mostly consisted of common factors related to delivery and management of MNCH services. A multivariate logistic regression analysis was performed between quality of care, socioeconomic factors, and service utilisation indicators among the intervention group participants who had at least one ANC visit.

**Results:**

Of the 3351 pregnant women who visited ANC at least once, 2911 without missing values among explanatory or dependent variables were included in the analysis. Among them, 2032 (69.8%) were exposed to optimal-quality ANC, and 2058 (70.7%), 1573 (54.0%), and 941 (32.3%) achieved ANC target, facility delivery, and vaccination target for six-month-old infants, respectively. Exposure to suboptimal-quality care at ANC was associated with lower odds for facility delivery (adjusted odds ratio (AOR) = 0.60, 95% CI = 0.49-0.73) and the achievement of the vaccination target (AOR = 0.43, 95% CI = 0.33-0.55). A low socioeconomic status was inversely associated with health service utilisation indicators.

**Conclusions:**

Health facilities’ quality of care influences subsequent MNCH service utilisation. Therefore, simultaneous efforts to improve quality of care and the mobilisation of pregnant women and communities are essential for enhancing maternal and child health.

Reduction in maternal, neonatal, and infant mortality is a critical issue in many low- and middle-income countries (LMICs); maternal and child mortality reduction is the highest priority of the United Nations Sustainable Development Goals among its health-related targets [1]. However, in many LMICs, especially in sub-Saharan African countries, maternal and child health mortality reduction target has not been achieved. Approximately two-thirds of maternal deaths globally occurred in sub-Saharan Africa in 2017 [2]. Additionally, according to World Bank data, 43% of neonatal deaths occurred in the region in 2019 [3]. Many evidence-based interventions have been introduced to prevent high maternal and child mortality in LMICs. However, their population-level impact depends not only on their efficacy, but also on their quality and reach to their target population [4,5].

Needs to improve health service utilisation of pregnant women and mothers have been repeatedly noted in LMICs. For example, in sub-Saharan Africa, 59% of pregnant women used four antenatal care (ANC) services, and 61% delivered their babies with support from skilled birth attendants. Additionally, 57% of the children received all childhood vaccinations [3,6,7]. One study found that mothers did not deliver children at a health facility in sub-Saharan Africa because they perceived that facility delivery was unnecessary [8]. Extensive epidemiological survey analyses, such as the Demographic and Health Survey, provide substantial evidence regarding the individual factors associated with maternal, newborn and child health (MNCH) service utilisation. A low socioeconomic status, such as a low income, limited education, being unmarried, and living in rural areas, have repeatedly been associated with poor MNCH service uptake [9-15]. To strengthen service utilisation, various efforts had been made in LMICs. Some strategies, such as providing reminders and incentives for service utilisation, proved effective in increasing service utilisation [16].

Improving quality of care is imperative in addition to increasing the quantity of service utilisation in LMICs [17,18]. Approximately five million deaths are estimated to occur every year in 137 LMICs owing to poor-quality care. The health burden surpasses that of inadequate service utilisation [18]. Quality of care refers to a wide range of issues, such as fidelity to guidelines or protocols, appropriate assessment and diagnosis, delivery of effective treatment, counseling, and user acceptance. Many of them can be understood as implementation problems. Health facilities’ readiness for MNCH services has been reported to be low. Health facility surveys demonstrated that there were large gaps between the recommendations and implementation status for some key ANC interventions [19]. An analysis of the Demographic and Health Survey exhibited low fidelity to the WHO recommendations on ANC in 10 sampled LMICs, even though the numbers of ANC visits reached a milestone of four [20-22]. One-third of people had negative experiences at health facilities; most felt that they were not treated with respect at maternity cares, an issue that has been around in LMICs for decades [18]. The evidence suggests that mere utilisation of such services is insufficient; the quality of care should be assured. Furthermore, quality of care is assumed to influence adherence to health services. One study in Ethiopia found that receiving appropriate content at ANC and the health facilities’ readiness for MNCH services were associated with better health service utilisation [10]. Furthermore, some studies found that community- and individual-level positive perceptions of health services were associated with an increased uptake of MNCH services [23,24]. Interventions strengthening health systems have demonstrated increased outpatient service utilisation, including facility delivery [25]. However, evidence regarding the impact of health facilities’ quality of care on MNCH service utilisation using population-based data are insufficient.

In a cluster randomised controlled trial to investigate the impact of the Maternal and Child Health Handbook (MCH-HB) on the continuum of care among pregnant women and mothers in Angola (MCH-HB RCT), implementation status was evaluated among the intervention group health facilities. The results illustrated that the quality of care offered during the trial differed widely among health facilities. Using the data set, this study investigated the influence of quality of care to which pregnant women were exposed on their subsequent MNCH service utilisation using population-based cohort data derived from the MCH-HB RCT [26-29]. To the best of our knowledge, this is the first study to demonstrate the association between quality of care and MNCH service utilisation using extensive population-based data.

## METHODS

### Study design

This study is a longitudinal analysis using a part of the data set of the MCH-HB RCT and its associated implementation study. Our research team conducted the MCH-HB RCT between June 2019 and September 2020 [26,27], and the implementation study between October and November 2020 [28,29].

### Study setting

The original MCH-HB RCT and implementation study were conducted in Benguela Province in Angola, a lower-middle-income country in sub-Saharan Africa [3]. Angola experiences high maternal and child deaths. The maternal mortality ratio was 241 per 100 000 live births in 2017 [2]. Neonatal and under-five mortality rates were 27 and 72, respectively, per 1000 live births in 2020 [3]. Slow improvement in maternal and child health indicators in Angola is partly due to poorly functioning health systems and insufficient health professionals [30]. According to the Demographic and Health Survey in 2015-2016 in Angola, the ANC receiving rate was 82%, the facility delivery rate was 54%, and the eight basic vaccine completion rate during the first year was 25% [31]. The Angolan government is trying to improve the continuum of care (CoC) among pregnant women, mothers, and their children, to address this situation.

The MCH-HB was introduced in Angola to increase the CoC as a national policy. Benguela is the third most populous province in Angola, with a population of approximately 2.2 million [32]. In five municipalities allocated to the intervention group in the MCH-HB RCT, health facilities providing MNCH services under the jurisdiction of the Ministry of Health implemented the MCH-HB program. The MCH-HB is an integrated home-based record. It registers key information such as health service utilisation and health conditions of mother and child from pregnancy to early childhood (e.g., maternal care and the child’s growth and immunisations) [33,34]. The MCH-HB program consisted of three components including distribution of the MCH-HB to pregnant women at health facilities, training of health workers on the MCH-HB operation, and community sensitisation targeting pregnant women on the MCH-HB use.

### Study population

The inclusion criteria of the MCH-HB RCT were (1) participant’s last menstrual period between 1 March and 30 April 2019 and (2) utilisation of maternal and child health services at public health facilities at least once. The exclusion criterion was the intention to move away from the study area. The original data source contained information on 9039 women for the primary analysis. This study analysed intervention-group participants who utilised ANC services at least once, their children alive until the follow-up survey and aged six months and older. Among the control group participants, the indicator of quality of care for health facilities was not obtained.

### Ethical approval

The original studies were approved by the ethical committee of National Center for Child Health and Development, Tokyo, Japan (No. 1721, 2020-026) and the Ethics Committee of the Ministry of Health of the Republic of Angola (protocol identification No. 15/2018, 768/DNSP/GD/2020). All the participants provided written consent.

### Measures

Individual socioeconomic factors, perceptions about ANC, and service utilisation were obtained from the MCH-HB RCT data set. The data were collected by interviewer-administered structured questionnaires. The indicators of quality of care for health facilities were obtained from the implementation study. The data were collected by structured questionnaires to health facilities and health workers, project operational records, and the MCH-HB RCT data set.

### Individual factors

Socioeconomic factors included age, marital status, educational level, wealth index, ethnicity, and residential area. Parity was an individual health history factor.

Age was divided into three categories: (1) 19 years or younger, (2) 20-34 years old, and (3) 35 years or older. Marital status was categorised into (1) married or cohabitating and (2) single, separated, or widowed. The educational level had three categories: (1) secondary education or more, (2) primary education, and (3) no formal education. The wealth index is a quintile index. Ethnicity was divided into two categories: (1) the biggest ethnic group (Umbundu) and (2) minor ethnic groups. The residential area by facility level was categorised into (1) urban and (2) rural. Finally, parity had two categories: (1) multiparous and (2) primiparous.

### User perception

User perception included six aspects of the users’ perception of ANC: (1) time of consultation, (2) sufficiency of information, (3) being treated with dignity and respect, (4) ability to discuss during consultations, (5) easy-to-understand information, and (6) follow-up scheduling.

The first and second items were assessed using a three-point scale between little and enough, and other items were assessed using a three-point scale between rarely and often.

### Service utilisation

The service utilisation indicators included optimal ANC, facility delivery, and optimal vaccinations for six-month-old. Optimal ANC represents the optimal uptake of ANC from the time of the first ANC services. To achieve this, women who first visited ANC at a maximum of 20 weeks of pregnancy were required to have four ANC visits before delivery. Those who visited an ANC service provider between 21 and 30 weeks of pregnancy were required to have three. Those between 31 and 37 weeks were required to have two, and those over 37 weeks were required to have one. Accordingly, optimal ANC had two categories: (1) “achieved” and (2) “not achieved”. The local target of four ANC visits was not used in this study as the focus is to understand the influence of health facilities’ quality of care on subsequent service utilisation. Facility delivery had two categories: (1) “achieved” and (2) “not achieved”. Finally, optimal vaccinations for six-month-old infants were assessed by the time points at which participants had their infants vaccinated. Four-time points before the infants were six months old were the target according to the local vaccination schedule. Thus, optimal vaccination at six months had two categories: (1) “achieved” and (2) “not achieved”.

### Health facilities’ quality of care

Among 89 health facilities in the MCH-HB RCT intervention group, a health facility’s quality of care was assessed using the MCH-HB implementation status. It indicates the facility’s capacity to launch the MCH-HB and achieve the required quality of care. The implementation of the MCH-HB was assessed mainly by common factors related to MNCH services and their management, described later in this section. Therefore, health facilities that achieved optimal implementation of the MCH-HB could provide basic MNCH services at optimal quality. Similarly, health facilities implementing the MCH-HB at a sub-optimal level were assessed as providing MNCH services at suboptimal quality. Accordingly, a health facility’s quality of care was categorised into (1) “optimal-quality” and (2) “suboptimal-quality care”. A health facility’s MCH-HB implementation status was a composite measure of 14 indicators defined by the discussion among researchers and the MCH-HB project specialists based on the MCH-HB program components and the RE-AIM framework to assess the implementation. The RE-AIM framework is an internationally used framework to evaluate implementation of public health intervention [4]. The 14 indicators are: (1) MCH-HB’s distribution coverage, (2) the facility’s representative’s participation in the training of the MCH-HB’s trainers, (3) delivery of the MCH-HB’s intra-facility training, (4) use of inventory management sheets, (5) regular delivery of mothers’ classes, (6) MCH-HB’s retention of the participants recruited from the facility, (7) appropriate birth weight completion on MCH-HB, (8) experience of MCH-HB’s stockout, (9) delivery of various themed mothers’ classes, (10) having a specific person responsible for MCH-HB training after the RCT’s term, (11) knowledge levels of health workers in management positions, (12) knowledge levels of health workers in non-management positions, (13) the burden of health workers in management positions, and (14) the burden of health workers in non-management positions. For each indicator, the target was predefined, and the indicator was converted to a binary variable. Achieving each indicator gave one point, and the total score ranges between 0 and 14. A score of nine or more or a 65% achievement rate was predefined as the threshold of optimal implementation [28,29]. Then, it was merged with the MCH-HB RCT’s data set using the facility codes.

### Statistical analysis

#### Descriptive analysis

Participants’ socioeconomic factors, past pregnancy, and service utilisation indicators were descriptively analysed and compared between those exposed to optimal- and suboptimal-quality care at ANC. For age, *t*-tests were performed. For variables besides age, χ^2^ tests were performed.

Analysis of the association between the indicator of quality of care for health facilities and user perceptions

The association with user perception of ANC was examined to assess the validity of the indicator of quality of care for health facilities. The differences in the six ANC user perception indicators between participants exposed to optimal-quality and suboptimal-quality care at ANC were analysed using χ^2^ tests.

### Logistic regression analysis

A logistic regression analysis was conducted between service utilisation indicators, the indicators for quality of care for health facilities, and individual factors. Optimal ANC, facility delivery, and optimal vaccinations for six-month-old infants were used as dependent variables. Health facility’s quality of care, and individual variables, including maternal age, marital status, maternal educational levels, wealth index, ethnicity, past pregnancy, and residential areas, were used as explanatory variables. For the analysis of facility delivery, optimal ANC was included in the set of explanatory variables. For the optimal vaccination analysis, optimal ANC and facility delivery were included.

First, the association between health service utilisation and explanatory variables was analysed using univariate logistic regression. The crude odds ratio was also calculated. Second, the association between health service utilisation indicators and all explanatory variables was analysed using multivariate logistic regression, and the adjusted odds ratio was calculated. The statistical significance threshold was set to *P* = 0.05. According to the original RCT definition, missing values for service utilisation indicators were imputed with the lowest achievement categories. Missing values for other variables were not imputed, as only participants without missing values were analysed. All analyses were conducted using R software, version 3.6.2.

### Stratified analysis

As service utilisation differed between urban and rural municipalities [27], baseline characteristics, health facilities’ quality of care and service utilisation indicators were descriptively analysed among urban and rural municipality participants. Afterwards, municipality-level stratified logistic regression analyses were conducted using the same explanatory and dependent variables. Among five municipalities allocated to the intervention group of the MCH-HB RCT, one municipality is categorised as urban, and four municipalities as rural.

## RESULTS

**Figure 1:**
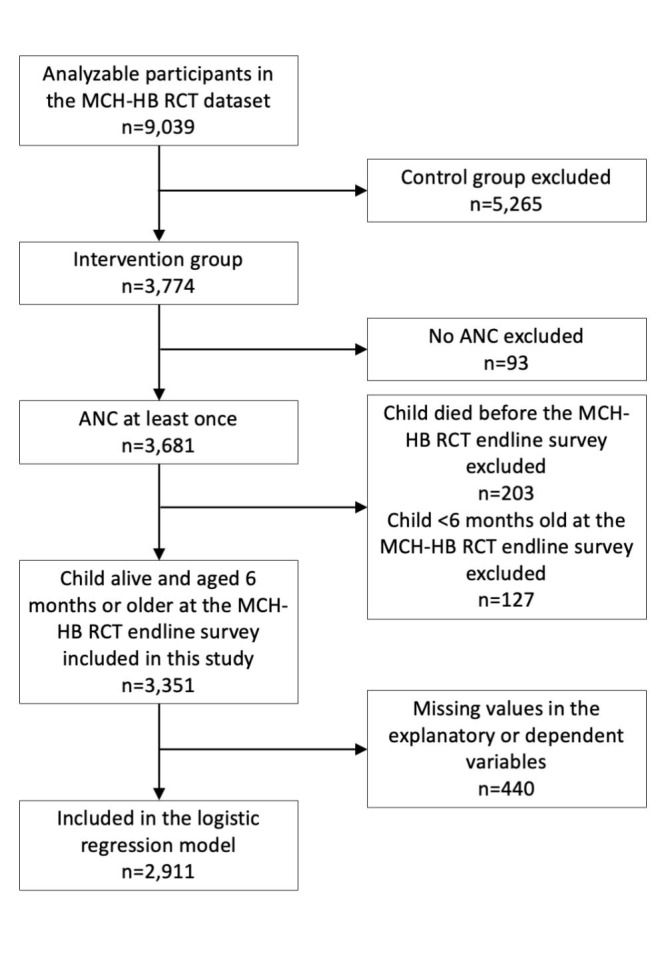
Flow of the participants included and analysed in this study.

Nine thousand and thirty-nine participants were analysed in the MCH-HB RCT, with 3774 participants allocated to the intervention group and 3681 utilising at least one ANC. Among those who utilised at least one ANC, 3351 participants whose children were alive and aged six months old or older at the follow-up survey were included in this study (**Figure 1**). The participants’ mean age was 24.7 years (standard deviation (SD) = 6.2). Among them, 2344 (69.9%), 1751 (52.3%), and 1027 (30.6%) achieved optimal ANC, facility delivery, and optimal vaccination with their six-month-old infants, respectively. Their characteristics are summarised in **Table 1**. Among 89 health facilities, 88 underwent the evaluation of the MCH-HB implementation status. The mean achievement rate of the implementation composite indicators was 63.3%, and 50 health facilities (56.8%) achieved the optimal implementation. Accordingly, 2243 (68.4%) received ANC at health facilities that provided optimal-quality care.

**Table 1:** Baseline characteristics and service utilization of participants and comparison between participants with and without missing values

	All participants (n = 3351)	Participants exposed to optimal-quality care* at ANC (n = 2243)	Participants exposed to suboptimal-quality care* at ANC (n = 1038)	*P*-value†
**Age, mean (SD)**	24.7 (6.2)	25.0 (6.3)	24.0 (6.1)	<0.001
20-34 y old	2241 (68.0%)	1543 (69.9%)	660 (64.7%)	<0.001
19 y or younger	774 (23.5%)	463 (21%)	287 (28.1%)	
35 y or more	282 (8.6%)	203 (9.2%)	73 (7.2%)	
**Marital status**				
Married or cohabitating	2277 (68.8%)	1548 (69.9%)	685 (66.8%)	0.08
Single, divorced, widowed	1033 (31.2%)	668 (30.1%)	341 (33.2%)	
**Maternal education**				
Secondary education or more	1358 (41.2%)	1054 (47.6%)	254 (25.1%)	<0.001
Primary education	1262 (38.3%)	763 (34.5%)	486 (48.0%)	
No formal education	674 (20.5%)	396 (17.9%)	273 (26.9%)	
**Wealth index**				
Wealthiest	506 (16.8%)	476 (23.2%)	5 (0.6%)	<0.001
Wealthy	514 (17.0%)	417 (20.3%)	71 (7.9%)	
Average	611 (20.2%)	377 (18.3%)	223 (24.8%)	
Poor	616 (20.4%)	386 (18.8%)	229 (25.5%)	
Poorest	773 (25.6%)	399 (19.4%)	370 (41.2%)	
**Ethnicity**				
The biggest ethnic group	2929 (88.9%)	1996 (90.7%)	871 (84.9%)	<0.001
Others	367 (11.1%)	205 (9.3%)	155 (15.1%)	
**Residential area classification (facility level)**				
Urban	1184 (35.4%)	893 (39.8%)	287 (27.6%)	<0.001
Rural	2160 (64.6%)	1350 (60.2%)	751 (72.4%)	
**Municipality classification**				
Urban	1435 (42.8%)	1206 (53.8%)	160 (15.4%)	<0.001
Rural	1916 (57.2%)	1037 (46.2%)	878 (84.6%)	
**Parity**				
Multiparous	2518 (76.1%)	1689 (76.2%)	787 (76.7%)	0.80
Primiparous	792 (23.9%)	527 (23.8%)	239 (23.3%)	
**Service utilization**				
Optimal ANC				
Not achieved	1007 (30.1%)	589 (26.3%)	408 (39.3%)	<0.001
Achieved	2344 (69.9%)	1654 (73.7%)	630 (60.7%)	
**Facility delivery**				
Not achieved	1600 (47.7%)	877 (39.1%)	710 (68.4%)	<0.001
Achieved	1751 (52.3%)	1366 (60.9%)	328 (31.6%)	
**Optimal vaccination**				
Not achieved	2324 (69.4%)	1363 (60.8%)	917 (88.3%)	<0.001
Achieved	1027 (30.6%)	880 (39.2%)	121 (11.7%)	

ANC – antenatal care, SD – standard deviation, y – year

*The indicators of quality of care were missing for 70 participants, so the total number of participants exposed to optimal- and suboptimal- quality of care does not equal the total number of participants.

†For comparison between participants exposed to optimal and suboptimal-quality care at ANC, a *t-*test was performed for a continuous variable (age), and a χ^2^ test was performed for categorical variables (variables other than age).

Compared to participants who were exposed to optimal-quality care at ANC, those who were exposed to suboptimal-quality care at ANC were more likely to be younger, less educated, poorer, belong to minority ethnic groups, living in rural residential areas, living in rural municipalities, and less achieving MNCH service utilisation targets (**Table 1**). Additionally, the indicator of quality of care for health facilities demonstrated a significant association between all six indicators regarding user perception of ANC (**Table 2**).

**Table 2:** Association between a health facility’s quality of care and user perceptions about ANC

	Optimal-quality care (n = 2243)	Suboptimal-quality care (n = 1038)	*P*-value*
**ANC consultation time**			
Little	561 (26.2%)	288 (29.5%)	0.004
Fair	580 (27.1%)	295 (30.2%)	
Enough	999 (46.7%)	393 (40.3%)	
**Sufficiency of ANC information**			
Little	251 (11.7%)	197 (19.7%)	<0.001
Fair	997 (46.4%)	443 (44.3%)	
Enough	902 (42.0%)	359 (35.9%)	
**ANC with dignity and respect**			
Rarely	279 (13.0%)	179 (17.9%)	<0.001
Sometimes	590 (27.4%)	377 (37.7%)	
Often	1281 (59.6%)	443 (44.3%)	
**Ability to discuss during ANC**			
Rarely	634 (29.5%)	391 (39.1%)	<0.001
Sometimes	693 (32.2%)	340 (34.0%)	
Often	823 (38.3%)	268 (26.8%)	
**ANC information easy to understand**			
Rarely	324 (15.1%)	252 (25.2%)	<0.001
Sometimes	679 (31.6%)	414 (41.4%)	
Often	1147 (53.3%)	333 (33.3%)	
**ANC follow-up scheduled**			
Rarely	221 (10.3%)	166 (16.6%)	<0.001
Sometimes	382 (17.8%)	347 (34.7%)	
Often	1547 (72.0%)	486 (48.6%)	

ANC – antenatal care

*χ^2^ tests were conducted to demonstrate the difference between participants exposed to ANC at optimal and suboptimal-quality care facilities.

### Logistic regression analysis

A total of 2911 participants without missing values among explanatory and dependent variables were included in the regression analyses (**Figure 1**). Among the explanatory variables, the variant inflation factor was <10. The highest was 1.87.

Univariate and multivariate logistic regression analyses were performed using the optimal ANC, facility delivery, and optimal vaccinations for six-month-old infants as the dependent variable. Univariate logistic regression analyses demonstrated statistically significant associations between the three service utilisation indicators and health facilities’ quality of care, maternal educational levels, wealth index, parity, and residential areas (Table S1 in the **Online Supplementary Document**). Teenage pregnancy and marital status were associated with a part of service utilisation indicators. Multivariate logistic regression analyses demonstrated that the health facility’s suboptimal-quality care was inversely associated with facility delivery and the achievement of optimal vaccination at a statistically significant level (facility delivery: adjusted odds ratio (AOR) = 0.60 (95% CI = 0.49-0.73), optimal vaccination: AOR = 0.43 (95% CI = 0.33-0.55)). The health facility’s quality of care was not significantly associated with optimal ANC (AOR = 0.89 (95% CI = 0.74-1.07)). Additionally, low maternal educational levels and lower economic levels remain significantly associated with poor service utilisation of all three service categories. Rural residential areas, being single, divorced or widowed, and past pregnancy were significantly associated with poor utilisation of a part of services. Optimal ANC and facility delivery were significantly associated with the subsequent service utilisation (**Table 3**).

**Table 3:** Adjusted odds ratio for optimal ANC, facility delivery, and optimal vaccination

	Optimal ANC*				Facility delivery				Optimal vaccination†			
	**Achieved (n = 2058)**	**Not achieved (n = 853)**	**AOR (95% CI)**	***P*-value**	**Achieved (n = 1573)**	**Not achieved (n = 1338)**	**AOR (95% CI)**	***P*-value**	**Achieved (n = 941)**	**Not achieved (n = 1970)**	**AOR (95% CI)**	***P*-value**
**Health facility's quality of care**												
Optimal‡	1507 (74.2%)	525 (25.8%)			1275 (62.7%)	757 (37.3%)			831 (40.9%)	1201 (59.1%)		
Suboptimal	551 (62.7%)	328 (37.3%)	0.89 (0.74-1.07)	0.22	298 (33.9%)	581 (66.1%)	0.60 (0.49-0.73)	<0.001	110 (12.5%)	769 (87.5%)	0.43 (0.33-0.55)	<0.001
**Maternal age group**												
20-34 y old‡	1433 (71.4%)	573 (28.6%)			1107 (55.2%)	899 (44.8%)			671 (33.4%)	1335 (66.6%)		
-19 y old	445 (67.9%)	210 (32.1%)	0.87 (0.67-1.12)	0.27	322 (49.2%)	333 (50.8%)	0.78 (0.59-1.03)	0.08	197 (30.1%)	458 (69.9%)	0.82 (0.61-1.10)	0.19
35 y old +	180 (72.0%)	70 (28.0%)	1.11 (0.81-1.51)	0.52	144 (57.6%)	106 (42.4%)	1.37 (0.99-1.90)	0.06	73 (29.2%)	177 (70.8%)	0.90 (0.63-1.27)	0.53
**Marital status**												
Married and / or cohabitating‡	1419 (70.1%)	604 (29.9%)			1027 (50.8%)	996 (49.2%)			602 (29.8%)	1421 (70.2%)		
Single, divorced, widowed	639 (72.0%)	249 (28.0%)	0.96 (0.77-1.18)	0.68	546 (61.5%)	342 (38.5%)	1.58 (1.26-1.99)	<0.001	339 (38.2%)	549 (61.8%)	1.05 (0.83-1.33)	0.70
**Maternal education**												
Secondary education or more‡	1004 (81.3%)	231 (18.7%)			966 (78.2%)	269 (21.8%)			655 (53.0%)	580 (47.0%)		
Primary education	705 (65.1%)	378 (34.9%)	0.78 (0.62-0.98)	0.03	449 (41.5%)	634 (58.5%)	0.55 (0.44-0.69)	<0.001	229 (21.1%)	854 (78.9%)	0.71 (0.56-0.90)	0.01
No formal education	349 (58.9%)	244 (41.1%)	0.68 (0.52-0.89)	0.01	158 (26.6%)	435 (73.4%)	0.42 (0.31-0.56)	<0.001	57 (9.6%)	536 (90.4%)	0.44 (0.31-0.63)	<0.001
**Wealth index**												
Wealthiest‡	434 (91.0%)	43 (9.0%)			448 (93.9%)	29 (6.1%)			339 (71.1%)	138 (28.9%)		
Wealthy	400 (83.0%)	82 (17.0%)	0.55 (0.37-0.82)	0.003	397 (82.4%)	85 (17.6%)	0.44 (0.28-0.69)	<0.001	259 (53.7%)	223 (46.3%)	0.71 (0.54-0.95)	0.02
Average	413 (70.2%)	175 (29.8%)	0.29 (0.20-0.43)	<0.001	337 (57.3%)	251 (42.7%)	0.17 (0.11-0.27)	<0.001	180 (30.6%)	408 (69.4%)	0.46 (0.34-0.62)	<0.001
Poor	371 (61.0%)	237 (39.0%)	0.21 (0.15-0.32)	<0.001	214 (35.2%)	394 (64.8%)	0.09 (0.06-0.14)	<0.001	82 (13.5%)	526 (86.5%)	0.23 (0.16-0.32)	<0.001
Poorest	440 (58.2%)	316 (41.8%)	0.20 (0.14-0.30)	<0.001	177 (23.4%)	579 (76.6%)	0.06 (0.04-0.09)	<0.001	81 (10.7%)	675 (89.3%)	0.25 (0.17-0.37)	<0.001
**Ethnicity**												
The biggest ethnic group‡	1843 (70.3%)	779 (29.7%)			1419 (54.1%)	1203 (45.9%)			843 (32.2%)	1779 (67.8%)		
Others	215 (74.4%)	74 (25.6%)	1.34 (0.9995-1.80)	0.054	154 (53.3%)	135 (46.7%)	1.16 (0.85-1.60)	0.352	98 (33.9%)	191 (66.1%)	1.22 (0.88-1.70)	0.24
**Residential area classification (facility based)**												
Urban‡	847 (76.9%)	255 (23.1%)			769 (69.8%)	333 (30.2%)			501 (45.5%)	601 (54.5%)		
Rural	1211 (66.9%)	598 (33.1%)	0.89 (0.73-1.08)	0.23	804 (44.4%)	1005 (55.6%)	0.67 (0.55-0.82)	<0.001	440 (24.3%)	1369 (75.7%)	0.71 (0.59-0.87)	<0.001
**Parity**												
Multiparous‡	1563 (69.7%)	678 (30.3%)			1169 (52.2%)	1072 (47.8%)			658 (29.4%)	1583 (70.6%)		
Primiparous	495 (73.9%)	175 (26.1%)	1.15 (0.87-1.51)	0.32	404 (60.3%)	266 (39.7%)	0.91 (0.68-1.23)	0.54	283 (42.2%)	387 (57.8%)	1.50 (1.12-2.02)	0.007
**Optimal ANC**												
Not achieved‡					277 (32.5%)	576 (67.5%)			122 (14.3%)	731 (85.7%)		
Achieved					1296 (63.0%)	762 (37.0%)	2.22 (1.82-2.70)	<0.001	819 (39.8%)	1239 (60.2%)	2.03 (1.59-2.58)	<0.001
**Facility delivery**												
Not achieved‡									156 (11.7%)	1182 (88.3%)		
Achieved									785 (49.9%)	788 (50.1%)	2.54 (2.01-3.20)	<0.001

ANC – antenatal care, AOR – adjusted odds ratio, CI – confidence interval, y – year

*Optimal ANC is defined by four or more ANC visits for women who first visited an ANC service provider at a maximum of 20 weeks of pregnancy, three or more ANC visits for those who visited an ANC service provider between 21 and 30 weeks of pregnancy, two or more ANC visits for those between 31 and 37 weeks, and once or more for those at 38 weeks and above.

†Optimal vaccination is defined by four or more vaccination by six-month postpartum.

‡Indicates reference categories.

### Stratified analyses analysis

Among 3351 included participants, 1435 and 1916 lived in urban and rural municipalities, respectively. Participants from rural municipalities were younger, less educated, poorer, likely to belong to a minority ethnic group, likely to receive suboptimal-quality care at ANC, and less achieved MNCH service utilisation targets. (Table S2 in the **Online Supplementary Document**) Stratified analyses among participants living in an urban municipality demonstrated that the health facility’s suboptimal-quality care was inversely associated with facility delivery and optimal vaccination (optimal ANC: AOR = 0.79 (95% CI = 0.51-1.23), facility delivery: AOR = 0.50 (95% CI = 0.32-0.78), optimal vaccination: AOR = 0.37 (95% CI = 0.18-0.75)), while those in rural municipalities, suboptimal-quality care was inversely associated with only optimal vaccination at a statistically significant level (optimal ANC: AOR = 1.15 (95% CI = 0.93-1.42), facility delivery: AOR = 0.92 (95% CI = 0.73-1.16), optimal vaccination: AOR = 0.55 (95% CI = 0.41-0.74)). The associations between maternal education, wealth index, and health service utilisation indicators were stronger in an urban municipality. Although residential areas were not significantly associated with any service utilisation indicators in an urban municipality, they were significantly associated with all three service utilisation indicators in rural municipalities. Most other explanatory variables demonstrated trends similar to the primary analysis, although some failed to reach statistical significance. (Table S3 and Table S4 in the **Online Supplementary Document**)

## DISCUSSION

This study analysed the association between the quality of care offered by health facilities to which pregnant women were exposed at ANC as well as subsequent MNCH service utilisation indicators among pregnant women who participated in the MCH-HB RCT. Overall, 2911 intervention group participants who used ANC services at least once were analysed. Women from lower socioeconomic groups were more likely to receive suboptimal-quality care at ANC. Exposure to ANC at suboptimal-quality care was negatively associated with facility delivery and the optimal vaccination at six months. Furthermore, key socioeconomic factors were also negatively associated with service utilisation indicators. Stratified analyses demonstrated that a health facility’s suboptimal-quality care is negatively associated with facility delivery and optimal vaccination in an urban municipality and with optimal vaccination in rural municipalities.

This study demonstrated strong associations between health facilities’ quality of care and the achievement of facility delivery as well as optimal vaccination for six-month-old infants using a data set from an extensive epidemiological study. The demonstrated associations are consistent with a few previous studies that discovered associations between quality of care indicators and MNCH service utilisation [10,35]. Although this study used an originally developed indicator as an indicator of quality of care, and those deployed in the previous studies were also varied, the association between exposure to optimal-quality MNCH services and improved subsequent service utilisation is reasonable and considered generalisable in LMICs. One significant reason women do not deliver at health facilities is that they perceive delivering at health facilities unnecessary; this is specifically true for sub-Saharan African countries [8]. However, women who received optimal-quality care might have felt that they received professional assessments and treatments with respect, and consequently understood the importance of MNCH services. This understanding may have encouraged women to complete ANC, deliver at health facilities, and get their children completely vaccinated. However, the association between quality of care and optimal ANC was not significant, possibly due to the relative ease of achieving this status, and the ambiguity of what it represented. Optimal ANC is a variable subjective to the timing of the first ANC; therefore, participants who visited ANC during late pregnancy achieved the target with only one ANC visit. However, they might have had negative attitudes toward MNCH services compared to those who visited ANC in earlier stages. The entire results suggest the need to improve the quality of care to increase service utilisation. Improvement in care quality improves the effectiveness and user fidelity to the MNCH service schedules. They would result in an increase in the health impact in a synergic way.

In addition to the associations between quality of care and subsequent MNCH service utilisation, this study replicated the known association between a low socioeconomic status and poor MNCH service utilisation [9-14]. In Angola, education, wealth, and residential areas were key socioeconomic indicators. Furthermore, the demonstrated difference between urban and rural municipalities might be explained by a few contextual issues. First, in rural municipalities in Angola, the baseline utilisation level of MNCH services was low. In particular, the delivery rate at facilities was lower owing to the barriers against facility delivery, such as challenging transition to distant health centers, lack of transport, among other obstacles. These barriers more significantly affected delivery than the quality of care. Second, vaccination was relatively strongly encouraged among MNCH services through community campaigns. This tendency might have caused a significant association with quality of care among rural municipality participants where vaccination was being established.

Furthermore, the results underlined the importance of evaluating how an intervention was delivered when we evaluated the impact of health interventions introduced to LMICs. Women exposed to suboptimal-quality care did not make expected behavioural changes, namely increased uptake of the subsequent MNCH services. In LMICs, health facilities often face obstacles in implementing interventions, and the quality of care differs considerably among providers. Additionally, the extent to which an intervention makes a change in health outcome depends on its quality. Therefore, reporting implementation indicators must be encouraged in all intervention studies, especially in LMICs. Further, to synthesise evidence regarding the effectiveness of health interventions in LMICs, their implementation must be taken into account. Otherwise, synthesised evidence cannot be understood appropriately and serve as a basis for policy-making.

### Implications for practice

Our results have a few implications for future practice. First, the results highlighted the need to assess the implementation of health interventions to evaluate their effectiveness in a global health context.

Second, the results suggested that improvement in quality of care directly benefited maternal and child health and indirectly benefited them through the increase in service utilisation. Hence, efforts to improve the quality of care and sensitise users should be simultaneously undertaken to promote maternal and child health in Angola. To bridge the gap in quality of care, health workers should be trained adequately before and during an intervention is introduced [28,36].

Third, the results suggest that pregnant women in low socioeconomic groups, especially those with lower education, on lower economic levels, and living in rural areas, require intensive support to utilise MNCH services at recommended levels. Strategies to reduce the barrier of service uptake include providing health education to increase the motivation as well as economic and transportation support for MNCH service utilisation for pregnant women, mothers, and families.

### Limitations

This study had some limitations. First, the causality between the exposure to suboptimal-quality care and subsequent incompletion of the service utilisation targets must be interpreted with caution as the temporality of the exposure and the outcome was not completely established. The implementation study was conducted at the end of the MCH-HB RCT so that the implementation status represented the quality of care at the end of the trial, but might not have represented that at the timing when participants visited ANC. However, the MCH-HB was implemented after health facilities acquired a certain level of knowledge and skills regarding its operation. Hence, during the study period, the health facility’s quality of care was considered as relatively consistent. Second, the indicator of quality of care utilised in this study was initially developed by our research team to examine MCH-HB implementation in health facilities [28]. No gold standard quality of care indicator exists for this research area. The quality indicator used in this study was a composite indicator that comprehensively evaluates an intervention’s delivery at a health facility. However, it shows reasonable variability and association with the facility’s location and category [28]. Hence, this indicator reasonably represents MNCH services’ quality of care. The study also demonstrated that a health facility’s optimal-quality care was significantly associated with users’ positive perceptions about ANC, which implies the appropriateness of the quality indicator used in this study. Third, the MCH-HB RCT recruited women who utilised MNCH services at least once. In particular, those who had negative experiences during previous pregnancies might decide not to utilise MNCH services any longer. Therefore, those women were not included in the present analysis. However, as those women are considered to be strongly influenced by quality of care, the current analysis does not overestimate the association between quality of care and service utilisation. Fourth, our study only analysed the participants with no missing values; therefore, 784 participants were omitted, undermining the results’ validity. However, considering the nature of extensive epidemiological surveys in LMICs, this data set’s proportion of missing values is acceptable.

## CONCLUSIONS

This study showed that health facilities’ quality of care influenced pregnant women’s subsequent MNCH service utilisation. The results demonstrated the importance of addressing implementation problems in LMICs and strengthening quality of care, in addition to sensitising pregnant women and communities, to improve maternal and child health in Angola.

## Additional material


Online Supplementary Document
