# Supplementary material for: Association between the quality of care and continuous maternal and child health service utilisation in Angola: Longitudinal data analysis
Source: J Glob Health. 2023 Aug 11;13:04073. doi: 10.7189/jogh.13.04073 (PMC10416139; doi:10.7189/jogh.13.04073)
Supplement: Online Supplementary Document [file jogh-13-04073-s001.pdf]

ONLINE SUPPLEMENTARY DOCUMENT

Title: Association between the quality of care and continuous maternal and child health service utilization in Angola: Longitudinal data analysis

Authors: Ai Aoki, Keiji Mochida, Michiru Kuramata, Toru Sadamori, Pedro Sapalalo, Lino Tchicondingosse, Olukunmi Omobolanle Balogun, Hirotsugu Aiga, Ketha Rubuz Francisco, Kenji Takehara

Table S1. Crude odds ratio for optimal ANC, facility delivery, and optimal vaccination

	**Optimal ANC***	**Facility delivery**	**Optimal vaccination†**
Health facility's quality of care			
Optimal‡			
Suboptimal	0.59 (0.49,0.69)	0.30 (0.26,0.36)	0.21 (0.17,0.26)
Maternal age group			
20-34 years old‡			
-19 years old	0.85 (0.70,1.03)	0.79 (0.66,0.94)	0.86 (0.71,1.04)
35 yeas old -	1.03 (0.77,1.38)	1.10 (0.85,1.44)	0.82 (0.62,1.09)
Marital status			
Married or cohabitating‡			
Single, divorced, widowed	1.09 (0.92,1.30)	1.55 (1.32,1.82)	1.46 (1.24,1.72)
Maternal education			
Secondary education or more‡			
Primary education	0.43 (0.35,0.52)	0.20 (0.16,0.24)	0.24 (0.20,0.29)
No formal education	0.33 (0.26,0.41)	0.10 (0.08,0.13)	0.09 (0.07,0.13)
Wealth index			
Wealthiest‡			
Wealthy	0.48 (0.33,0.72)	0.30 (0.19,0.47)	0.47 (0.36,0.62)
Average	0.23 (0.16,0.34)	0.09 (0.06,0.13)	0.18 (0.14,0.23)
Poor	0.16 (0.11,0.22)	0.04 (0.02,0.05)	0.06 (0.05,0.09)
Poorest	0.14 (0.10,0.19)	0.02 (0.01,0.03)	0.05 (0.04,0.07)
Ethnicity			
The biggest ethnic group‡			

Others	1.23 (0.93,1.62)	0.97 (0.76,1.23)	1.08 (0.84,1.40)
Residential area classification (facility level)			
Urban‡			
Rural	0.61 (0.51,0.72)	0.35 (0.30,0.41)	0.39 (0.33,0.45)
Parity			
Multiparous‡			
Primiparous	1.23 (1.01,1.49)	1.39 (1.17,1.66)	1.76 (1.47,2.10)
Optimal ANC			
Not achieved‡			
Achieved		3.54 (2.99,4.19)	3.96 (3.21,4.89)
Facility delivery			
Not achieved‡			
Achieved			7.55 (6.22,9.16)

ANC – antenatal care

*Optimal ANC is defined by four or more ANC visits for women who first visited an ANC service provider at a maximum of 20 weeks of pregnancy, three or more ANC visits for those who visited an ANC service provider between 21 and 30 weeks of pregnancy, two or more ANC visits for those between 31 and 37 weeks, and once or more for those at 38 weeks and above.

†Optimal vaccination is defined by four or more vaccination by six-month postpartum.

‡Indicates reference categories.

Table S2. Comparison between participants from urban and rural municipalities

	**Urban municipality (n=1,435)**	**Rural municipalities (n= 1,916)**	**P value**
Age (mean, SD)	25.1 (6.1)	24.3 (6.3)	<0.001
20-34 years old	1013 (71.3%)	1228 (65.4%)	<0.001
19 years or younger	288 (20.3%)	486 (25.9%)	
35 yeas or older	119 (8.4%)	163 (8.7%)	
Marital status			
Married and/or cohabitating	978 (68.7%)	1299 (68.8%)	0.98
Single, divorced, widowed	445 (31.3%)	588 (31.2%)	

Maternal education			
Secondary education or more	834 (59%)	524 (27.9%)	<0.001
Primary education	394 (27.9%)	868 (46.1%)	
No formal education	185 (13.1%)	489 (26%)	
Wealth index			
Wealthiest	482 (34.7%)	24 (1.5%)	<0.001
Wealthy	370 (26.6%)	144 (8.8%)	
Average	216 (15.5%)	395 (24.2%)	
Poor	159 (11.4%)	457 (28.0%)	
Poorest	163 (11.7%)	610 (37.4%)	
Ethnicity			
The biggest ethnic group	1284 (90.4%)	1645 (87.7%)	0.02
Others	137 (9.6%)	230 (12.3%)	
Residential area classification (facility based)			
Urban	451 (31.6%)	733 (38.3%)	<0.001
Rural	977 (68.4%)	1183 (61.7%)	
Parity			
Multiparous	1077 (75.7%)	1441 (76.4%)	0.68
Primiparous	346 (24.3%)	446 (23.6%)	
Health facility's quality of care			
Optimal	1206 (88.3%)	1037 (54.2%)	<0.001
Suboptimal	160 (11.7%)	878 (45.8%)	
Health service utilization			
Optimal ANC			
Not achieved	244 (17.0%)	763 (39.8%)	<0.001
Achieved	1191 (83.0%)	1153 (60.2%)	
Facility delivery			
Not achieved	344 (24.0%)	1256 (65.6%)	<0.001
Achieved	1091 (76.0%)	660 (34.4%)	
Optimal vaccination			
Not achieved	743 (51.8%)	1581 (82.5%)	<0.001

Achieved	692 (48.2%)	335 (17.5%)	

ANC – antenatal care, SD – standard deviation

* For comparison between participants from urban and rural municipalities, a t-test was performed for a continuous variable (age), and a chi-squared test was performed for categorical variables (variables other than age).

Table S3. Adjusted odds ratio for optimal ANC, facility delivery, and optimal vaccination in urban municipalities

	**Optimal ANC***				**Facility delivery**				**Optimal vaccination†**			
	**Achieved n (%)**	**Not achieved n (%)**	**AOR (95% CI)**	**P value**	**Achieved n (%)**	**Not achieved n (%)**	**AOR (95% CI)**	**P value**	**Achieved n (%)**	**Not achieved n (%)**	**AOR (95% CI)**	**P value**
Health facility's quality of care												
Optimal‡	992 (85.2%)	172 (14.8%)			948 (81.4%)	216 (18.6%)			631 (54.2%)	533 (45.8%)		
Suboptimal	93 (66.9%)	46 (33.1%)	0.79 (0.51,1.23)	0.30	45 (32.4%)	94 (67.6%)	0.50 (0.32,0.78)	0.003	11 (7.9%)	128 (92.1%)	0.37 (0.18,0.75)	0.01
Maternal age group												
20-34 years old‡	780 (83.2%)	158 (16.8%)			734 (78.3%)	204 (21.7%)			477 (50.9%)	461 (49.1%)		
19 years or younger	213 (83.2%)	43 (16.8%)	1.05 (0.66,1.66)	0.85	168 (65.6%)	88 (34.4%)	0.55 (0.34,0.88)	0.01	121 (47.3%)	135 (52.7%)	0.89 (0.58,1.37)	0.59
35 yeas or older	92 (84.4%)	17 (15.6%)	1.16 (0.65,2.06)	0.62	91 (83.5%)	18 (16.5%)	1.61 (0.86,3.04)	0.14	44 (40.4%)	65 (59.6%)	0.65 (0.41,1.04)	0.07
Marital status												
Married or cohabitating‡	737 (82.3%)	159 (17.7%)			675 (75.3%)	221 (24.7%)			424 (47.3%)	472 (52.7%)		
Single, divorced, widowed	348 (85.5%)	59 (14.5%)	1.09 (0.73,1.61)	0.68	318 (78.1%)	89 (21.9%)	1.25 (0.83,1.89)	0.29	218 (53.6%)	189 (46.4%)	0.86 (0.61,1.23)	0.42
Maternal education												
Secondary education or more‡	685 (89.5%)	80 (10.5%)			695 (90.8%)	70 (9.2%)			502 (65.6%)	263 (34.4%)		
Primary education	282 (77.5%)	82 (22.5%)	0.72 (0.47,1.10)	0.13	226 (62.1%)	138 (37.9%)	0.48 (0.31,0.72)	<0.001	118 (32.4%)	246 (67.6%)	0.71 (0.5,1.002)	0.0503
No formal education	118 (67.8%)	56 (32.2%)	0.61 (0.36,1.03)	0.07	72 (41.4%)	102 (58.6%)	0.36 (0.21,0.60)	<0.001	22 (12.6%)	152 (87.4%)	0.43 (0.24,0.78)	0.01
Wealth index												
Wealthiest‡	415 (91.6%)	38 (8.4%)			433 (95.6%)	20 (4.4%)			322 (71.1%)	131 (28.9%)		

Wealthy	303 (89.1%)	37 (10.9%)	0.86 (0.52,1.41)	0.55	303 (89.1%)	37 (10.9%)	0.54 (0.30,0.97)	0.04	205 (60.3%)	135 (39.7%)	0.79 (0.57,1.10)	0.16
Average	155 (77.1%)	46 (22.9%)	0.41 (0.24,0.69)	<0.001	142 (70.6%)	59 (29.4%)	0.24 (0.13,0.45)	<0.001	86 (42.8%)	115 (57.2%)	0.66 (0.43,1.004)	0.051
Poor	117 (74.1%)	41 (25.9%)	0.40 (0.22,0.74)	0.004	70 (44.3%)	88 (55.7%)	0.11 (0.06,0.21)	<0.001	18 (11.4%)	140 (88.6%)	0.15 (0.08,0.29)	<0.001
Poorest	95 (62.9%)	56 (37.1%)	0.26 (0.14,0.49)	<0.001	45 (29.8%)	106 (70.2%)	0.07 (0.04,0.14)	<0.001	11 (7.3%)	140 (92.7%)	0.15 (0.07,0.33)	<0.001
Ethnicity												
The biggest ethnic group‡	975 (82.8%)	202 (17.2%)			878 (74.6%)	299 (25.4%)			559 (47.5%)	618 (52.5%)		
Others	110 (87.3%)	16 (12.7%)	1.08 (0.61,1.92)	0.78	115 (91.3%)	11 (8.7%)	2.90 (1.39,6.08)	0.01	83 (65.9%)	43 (34.1%)	1.51 (0.96,2.38)	0.08
Residential area classification (facility based)												
Urban‡	384 (90.1%)	42 (9.9%)			395 (92.7%)	31 (7.3%)			282 (66.2%)	144 (33.8%)		
Rural	701 (79.9%)	176 (20.1%)	0.93 (0.61,1.43)	0.74	598 (68.2%)	279 (31.8%)	0.81 (0.50,1.31)	0.40	360 (41.0%)	517 (59.0%)	1.007 (0.75,1.36)	0.96
Parity												
Multiparous‡	816 (81.8%)	181 (18.2%)			749 (75.1%)	248 (24.9%)			448 (44.9%)	549 (55.1%)		
Primiparous	269 (87.9%)	37 (12.1%)	1.18 (0.72,1.95)	0.51	244 (79.7%)	62 (20.3%)	0.86 (0.51,1.46)	0.57	194 (63.4%)	112 (36.6%)	1.83 (1.19,2.81)	0.01
Optimal ANC												
Not achieved‡					112 (51.4%)	106 (48.6%)			37 (17.0%)	181 (83.0%)		
Achieved					881 (81.2%)	204 (18.8%)	2.41 (1.65,3.51)	<0.001	605 (55.8%)	480 (44.2%)	3.74 (2.46,5.69)	<0.001
Facility delivery												
Not achieved‡									45 (14.5%)	265 (85.5%)		
Achieved									597 (60.1%)	396 (39.9%)	2.64 (1.75,3.97)	<0.001

ANC – antenatal care, AOR –adjusted odds ratio, CI – confidence interval

*Optimal ANC is defined by four or more ANC visits for women who first visited an ANC service provider at a maximum of 20 weeks of pregnancy, three or more ANC visits for those who visited an ANC service provider between 21 and 30 weeks of pregnancy, two or more ANC visits for those between 31 and 37 weeks, and once or more for those at 38 weeks and above.

†Optimal vaccination is defined by four or more vaccination by six-month postpartum.

‡Indicates reference categories.

Table S4. Adjusted odds ratio for optimal ANC, facility delivery, and optimal vaccination in rural municipalities

	**Optimal ANC***				**Facility delivery**				**Optimal vaccination†**			
	**Achieved n (%)**	**Not achieved n (%)**	**AOR (95% CI)**	**P value**	**Achieved n (%)**	**Not achieved n (%)**	**AOR (95% CI)**	**P value**	**Achieved n (%)**	**Not achieved n (%)**	**AOR (95% CI)**	**P value**
Health facility's quality of care												
Optimal‡	515 (59.3%)	353 (40.7%)			327 (37.7%)	541 (62.3%)			200 (23.0%)	668 (77.0%)		
Suboptimal	458 (61.9%)	282 (38.1%)	1.15 (0.93,1.42)	0.19	253 (34.2%)	487 (65.8%)	0.92 (0.73,1.16)	0.48	99 (13.4%)	641 (86.6%)	0.55 (0.41,0.74)	<0.001
Maternal age group												
20-34 years old‡	653 (61.1%)	415 (38.9%)			373 (34.9%)	695 (65.1%)			194 (18.2%)	874 (81.8%)		
19 years or younger	232 (58.1%)	167 (41.9%)	0.81 (0.59,1.11)	0.19	154 (38.6%)	245 (61.4%)	0.91 (0.65,1.30)	0.62	76 (19.0%)	323 (81.0%)	0.79 (0.52,1.21)	0.28
35 yeas or older	88 (62.4%)	53 (37.6%)	1.12 (0.77,1.62)	0.55	53 (37.6%)	88 (62.4%)	1.37 (0.91,2.05)	0.13	29 (20.6%)	112 (79.4%)	1.34 (0.82,2.17)	0.24
Marital status												
Married or cohabitating‡	682 (60.5%)	445 (39.5%)			352 (31.2%)	775 (68.8%)			178 (15.8%)	949 (84.2%)		
Single, divorced, widowed	291 (60.5%)	190 (39.5%)	0.83 (0.64,1.08)	0.17	228 (47.4%)	253 (52.6%)	1.67 (1.25,2.22)	<0.001	121 (25.2%)	360 (74.8%)	1.26 (0.89,1.78)	0.19

Maternal education												
Secondary education or more‡	319 (67.9%)	151 (32.1%)			271 (57.7%)	199 (42.3%)			153 (32.6%)	317 (67.4%)		
Primary education	423 (58.8%)	296 (41.2%)	0.83 (0.63,1.09)	0.18	223 (31.0%)	496 (69.0%)	0.60 (0.45,0.79)	<0.001	111 (15.4%)	608 (84.6%)	0.75 (0.53,1.04)	0.09
No formal education	231 (55.1%)	188 (44.9%)	0.73 (0.53,1.01)	0.06	86 (20.5%)	333 (79.5%)	0.49 (0.34,0.7)	<0.001	35 (8.4%)	384 (91.6%)	0.51 (0.31,0.82)	0.006
Wealth index												
Wealthiest‡	19 (79.2%)	5 (20.8%)			15 (62.5%)	9 (37.5%)			17 (70.8%)	7 (29.2%)		
Wealthy	97 (68.3%)	45 (31.7%)	0.57 (0.20,1.64)	0.30	94 (66.2%)	48 (33.8%)	1.49 (0.58,3.81)	0.41	54 (38.0%)	88 (62.0%)	0.27 (0.10,0.73)	0.01
Average	258 (66.7%)	129 (33.3%)	0.58 (0.21,1.61)	0.30	195 (50.4%)	192 (49.6%)	1.05 (0.43,2.57)	0.92	94 (24.3%)	293 (75.7%)	0.19 (0.07,0.51)	<0.001
Poor	254 (56.4%)	196 (43.6%)	0.45 (0.16,1.26)	0.13	144 (32.0%)	306 (68.0%)	0.70 (0.28,1.72)	0.43	64 (14.2%)	386 (85.8%)	0.15 (0.06,0.41)	<0.001
Poorest	345 (57.0%)	260 (43.0%)	0.50 (0.18,1.40)	0.19	132 (21.8%)	473 (78.2%)	0.48 (0.19,1.20)	0.12	70 (11.6%)	535 (88.4%)	0.18 (0.07,0.49)	<0.001
Ethnicity												
The biggest ethnic group‡	868 (60.1%)	577 (39.9%)			541 (37.4%)	904 (62.6%)			284 (19.7%)	1161 (80.3%)		
Others	105 (64.4%)	58 (35.6%)	1.48 (1.04,2.11)	0.03	39 (23.9%)	124 (76.1%)	0.89 (0.58,1.35)	0.57	15 (9.2%)	148 (90.8%)	0.86 (0.47,1.56)	0.62
Residential area classification (facility level)												
Urban‡	463 (68.5%)	213 (31.5%)			374 (55.3%)	302 (44.7%)			219 (32.4%)	457 (67.6%)		
Rural	510 (54.7%)	422 (45.3%)	0.60 (0.48,0.76)	<0.001	206 (22.1%)	726 (77.9%)	0.40 (0.31,0.51)	<0.001	80 (8.6%)	852 (91.4%)	0.37 (0.27,0.51)	<0.001
Parity												
Multiparous‡	747 (60.0%)	497 (40.0%)			420 (33.8%)	824 (66.2%)			210 (16.9%)	1034 (83.1%)		
Primiparous	226 (62.1%)	138 (37.9%)	1.26 (0.90,1.76)	0.19	160 (44.0%)	204 (56.0%)	1.0041 (0.6952,1.4502)	0.98	89 (24.5%)	275 (75.5%)	1.22 (0.79,1.89)	0.36

Optimal ANC												
Not achieved‡					165 (26.0%)	470 (74.0%)			85 (13.4%)	550 (86.6%)		
Achieved					415 (42.7%)	558 (57.3%)	1.83 (1.44,2.32)	<0.001	214 (22.0%)	759 (78.0%)	1.31 (0.97,1.77)	0.08
Facility delivery												
Not achieved‡									111 (10.8%)	917 (89.2%)		
Achieved									188 (32.4%)	392 (67.6%)	2.20 (1.63,2.97)	<0.001

ANC – antenatal care, AOR –adjusted odds ratio, CI – confidence interval

*Optimal ANC is defined by four or more ANC visits for women who first visited an ANC service provider at a maximum of 20 weeks of pregnancy, three or more ANC visits for those who visited an ANC service provider between 21 and 30 weeks of pregnancy, two or more ANC visits for those between 31 and 37 weeks, and once or more for those at 38 weeks and above.

†Optimal vaccination is defined by four or more vaccination by six-month postpartum.

‡Indicates reference categories.
